# Assessing hygiene and zoonotic risk perception in the Danube Delta: insights from the pre- and early-COVID-19 periods

**DOI:** 10.3389/fvets.2026.1794566

**Published:** 2026-06-24

**Authors:** Mihai Marinov, Angela M. Ionică, Daniel N. Maftei, Lucian E. Bolboaca, Alexandru Dorosencu, Marian Tudor, Vasile Alexe

**Affiliations:** 1 Department of Biodiversity Conservation and Sustainable Use of Natural Resources, Danube Delta National Institute for Research and Development, Tulcea, Romania; 2 Clinical Hospital of Infectious Diseases of Cluj-Napoca, Cluj-Napoca, Romania; 3 Tulcea County Sanitary Veterinary and Food Safety Directorate, Tulcea, Romania; 4 Center for the Study of Trans-Border and Emerging Diseases and Zoonoses, Danube Delta National Institute for Research and Development, Tulcea, Romania; 5 Danube Delta National Institute for Research and Development, Tulcea, Romania

**Keywords:** awareness, COVID-19, Danube Delta, prevention, public health, risks, zoonoses

## Abstract

Zoonoses, or diseases that are naturally transmitted between animals and humans, are a global public health problem, accounting for over 60% of all infectious and parasitic diseases affecting humans. Therefore, raising awareness of the risks within communities is an activity that must be constant. The issue of zoonoses is of particular importance in regions characterized by high biodiversity, such as the Danube Delta. In this complex ecosystem, the frequency of interactions between the human population, native wildlife, and domestic animals creates favorable conditions for the transmission and spread of zoonotic pathogens, thus amplifying the risks to public health in the area. Despite information campaigns organized by local veterinary and public health authorities to educate communities about zoonotic diseases, feedback on the uptake of this information among residents remains limited. We used two cross-sectional waves (pre-COVID-19, 2019; COVID-19 onset, 2020), administering a questionnaire with 45 items in five domains (context, domestic animals, wildlife hazards, foodborne pathogens, and ticks) to assess awareness levels about zoonoses among respondents in 23 localities in Tulcea County, adjacent to the Danube Delta Biosphere Reserve (DDBR). Descriptive statistics (proportion with 95% Cls) and year-to-year comparisons (chi-square tests) were conducted. A total of 292 adults provided complete or partial responses, 157 respondents in 2019, and 135 in 2020. The results indicated that although the majority of respondents demonstrated a fairly good understanding of zoonotic diseases and applied a range of prevention measures, there are still notable gaps in knowledge and risk-related behaviors, exemplified by the differential trust and cognitive dissonance observed. The emergence of COVID-19 has further highlighted the importance of addressing zoonotic threats and reinforced the role of veterinarians as vital communicators in public health education. Therefore, it is imperative to implement more information campaigns aimed at correcting existing knowledge deficits, improving awareness, and applying prevention measures. Continued community engagement efforts are essential to strengthening public understanding of zoonotic diseases and facilitating effective prevention strategies.

## Introduction

Zoonoses were initially defined, according to World Health Organization (1), as diseases and infections that are naturally transmitted between vertebrate animals and humans. However, there are also invertebrate diseases that can be transmitted to humans. These can be transmitted to humans from an infected vertebrate host either through direct contact or indirectly—through ingestion of contaminated water or food, mechanical or biological vectors, or contact with contaminated items (2). Over 200 types of zoonotic diseases are currently recognized, accounting for a large proportion of human diseases, according to the World Health Organization (3). It is estimated that more than 60% of all human infectious diseases originate from animals (4). Several emerging infectious diseases also originate from wildlife, and the rise of these diseases is often driven by the dynamic interactions among wildlife, domestic animals, and human populations, in addition to rapidly changing environments (5, 6). COVID-19 exemplifies a zoonotic spillover event, reinforcing the need for One Health approaches that integrate human, animal, and environmental health to monitor, prevent, and respond to health threats across contexts.

This study was initiated in 2019 to assess progress in raising awareness of and implementing warnings issued by veterinary and public health authorities regarding the development of multiple outbreaks of avian influenza and African swine fever in the Danube Delta area; however, due to the evolution of COVID-19 (the first case in Romania was reported at the end of February 2020) and the popularized preventive measures, the hypothesis emerged of a better understanding by the population about the role of hygiene and prevention measures recommended for other diseases. Data were collected in 2019–2020; we acknowledge the delay in publication. Although initial results seemed insufficient for publication, subsequent regional outbreaks and deteriorating relations between authorities and livestock producers increased the study’s relevance. We therefore present these findings as a pre-event baseline and hypothesis-generating data, noting temporal limitations and calling for follow-up longitudinal studies.

Although the mechanisms of the emergence and/or adaptation of zoonoses are complex, many studies have identified geoclimatic factors and global climate change as important drivers of the current global disease burden (7). Zoonotic disease epidemiology is influenced by geoclimatic variations through alterations in host, vector, and pathogen dynamics and their interactions. However, modifications in the lifecycles of vectors, reservoirs, and pathogens, along with diseases of domestic and wild animals, are influenced by multiple complex processes (8). Climate change is occurring alongside environmental and landscape use changes, demographic changes, globalization, and increases in international trade and mobility; these factors also act as drivers and often cannot be delineated or separated (9). The Danube Delta Biosphere Reserve (DDBR) is located in eastern Romania, covers a total of 5,800 km^2^ (mainly in the territory of Tulcea County), and includes 30 types of ecosystems. It is one of the most important stopover sites for migratory birds, with representatives of over 300 avian species annually using the region’s ecosystems (10). In biodiversity-rich regions such as the Danube Delta, human-animal interfaces—encompassing domestic animals, wildlife, and livelihoods such as agriculture, fishing, and tourism—create complex exposure pathways that can shape risk perceptions and preventive behaviors among residents. Occupational exposures related to agriculture, aquaculture, birdwatching, and interactions with wildlife can further influence adherence to hygiene messages and the effectiveness of public health campaigns aimed at preventing zoonotic diseases, especially given the variable access to water, sanitation, and hygiene (WASH) in these specific conditions.

Public compliance with food hygiene, personal hygiene, and individual protection was analyzed in a global framework, indicating that the majority of respondents adopted the recommended measures. However, there were significant gaps observed (e.g., the use of masks in public spaces, incorrect hand washing/disinfection) requiring more effective risk communication (11). In parallel, studies in the United States have shown that the frequency of hand washing/disinfection is generally high but varies according to demographic factors and risk perception, suggesting the need for specific campaigns targeting groups such as men and young adults (12). These observations highlight that the impact of the “pandemic” on the population depends on risk behavior rather than on the availability of protective resources.

Analyses of population behavior in the context of fear and risk perception show that adherence is influenced by personal concerns and experiences with COVID-19, and communication messages should be adapted to increase hygiene compliance in public spaces and households (12).

The same is true for the population of the Danube Delta, especially for those who raise animals and live in isolation and sometimes in poor hygienic conditions. Unfortunately, they are more easily exposed online to disruptive influences - such as various conspiracy theories - that favor negative reactions to the measures imposed by the authorities and increase the risk of disease control and public health issues. In the study area, the Tulcea County Public Health Authority (CPHA) and the Tulcea Sanitary-Veterinary and Food Safety Directorate (TSVFSD), in particular, organize warning and informational campaigns regarding infectious diseases. However, there is no feedback regarding the assimilation of the provided information by the locals or visiting tourists. The two-wave cross-sectional design captures pre-pandemic baseline values and early pandemic dynamics, although we are aware of limitations in causal inference due to sampling and potential response bias.

Therefore, the present study aimed to assess the awareness level of the risks of animal-to-human transmission of zoonotic diseases among individuals living in the DDBR and surrounding areas by identifying channels and barriers to information uptake.

## Methods

The objective of this study was to characterize knowledge of, risk perception of, and hygiene practices related to zoonotic diseases, along with the impact of early communications about COVID-19 on these factors in 23 localities in Tulcea County, Romania, which is adjacent to the Danube Delta Biosphere Reserve (DDBR). The region features mixed, rural, and agricultural livelihoods, variable access to water, sanitation, and hygiene (WASH) resources, and close human-animal interfaces (domestic and wild animals) that may influence risk perception and preventive behavior.

We conducted two cross-sectional surveys (pre-COVID-19, Sept–Oct 2019; early-COVID-19, Sept–Oct 2020, respectively) using self-completed printed questionnaires developed in collaboration with veterinarians from the local veterinary authority. This questionnaire was designed to capture the knowledge and practices of individuals with direct or indirect contact with birds and wild animals, either permanently or in transit, through the DDBR. The questionnaire was written in Romanian, which is spoken and understood by all the different ethnic groups in the area. Eligible participants were 18 years of age or older, with direct or indirect contact with birds or wildlife as part of their daily lives (e.g., farming, fishing, birdwatching, or tourism).

Ethical approval for the study was obtained from the Danube Delta National Institute (DDNI) Scientific Council Approval Committee, which was at that time the equivalent of a Research Ethics Committee, with approval numbers 5 on 14 March 2019, and 6 on 1 April 2020.

Informed written consent was obtained from all interviewed participants, along with the distribution of the questionnaires.

The first investigations began in the second part of September and early October 2019. The following year, the COVID-19 pandemic evolved, and we considered it necessary to apply the same questionnaire in September and October to assess whether the recommended hygiene and movement restrictions during the pandemic brought improvements in how this population, constantly exposed to the risks related to animal husbandry and wildlife, understood to modify their behaviors and certain habits closely related to the activities they carry out. Written questionnaires were randomly distributed to individuals in 23 localities in Tulcea County, located in or near the DDBR. The questionnaire comprised five principal sections: (i) background information (9 items addressing occupation, awareness, personal hygiene, and children’s sanitary education); (ii) domestic animals (13 items concerning contact, care, hygiene, and prophylactic measures); (iii) wildlife-related hazards (9 items); (iv) foodborne pathogens (7 items); and (v) ticks (7 items), for a total of 45 questions.

Data were checked upon entry; incomplete questionnaires were retained as partial responses to maximize the sample size. We acknowledge the potential for social-desirability bias, limited qualitative insight due to the small number of open responses, and substantial non-response for some items, which may bias year-to-year comparisons. Paper responses were double-entered into a secure database, with access restricted to the research team, and were backed up regularly.

The maps were created in QGIS (version 3.22.4-Białowieża) using the Corine Land-Cover (2018) classes (32).

The responses provided by the interviewees are recorded in Tables 1–6 and were then introduced into Microsoft Excel spreadsheets and analyzed using EpiInfo 7 software (CDC, USA). The frequency of each type of response and the 95% Confidence Interval (CI) were calculated for each question, and differences between the two study years were assessed using chi-square testing and considered significant at *p* < 0.05.

**Table 1:** The localities where the questionnaire respondents live.

Locality	2019	2020	Total
Agighiol	1	1	2
Babadag	–	2	2
Balabancea*	–	1	1
Bucuresti*	–	1	1
Colina	2	–	2
Constanța*	–	1	1
Dorna Arini*	–	1	1
Dunavăț	–	1	1
Enisala	1	–	1
Iazurile	13	6	19
Ilgani	2	–	2
Isaccea	12	–	12
Jurilovca	20	10	30
Malcoci	1	–	1
Maliuc	14	12	26
Mineri	1	–	1
Murighiol	–	1	1
Niculițel	20	19	39
Nufărul	–	1	1
Partizani	16	–	16
Ploiești*	–	3	3
Sălcioara	20	13	33
Tulcea	26	43	69
Valea Nucarilor	5	1	6
Vișina	1	1	2
Vulturu	–	5	5
NA	2	12	14
Total	157	135	292

*Indicates areas beyond the Danube Delta Biosphere Reserve.

**Table 2:** The distribution of answers to questions in the first section (1–9).

Item	*n* (%; 95% CI)		*p*-value (numbers)
	2019	2020	
Occupation			
None	28 (17.83; 12.19–24.73)	10 (7.41; 3.61–13.2)	0.008
Employed (not specified)	13 (8.28; 4.48–13.74)	7 (5.19; 2.11–10.39)	0.296
Research	18 (11.46; 6.94–17.51)	11 (8.15; 4.14–14.11)	0.345
Agriculture or animal-related	37 (23.57; 17.17–30.99)	41 (30.37; 22.76–38.86)	0.190
Other	45 (28.66; 21.74–36.41)	43 (31.85; 24.1–40.42)	0.554
Retired	12 (7.64; 4.01–12.97)	16 (11.85; 6.93–18.53)	0.223
No answer	4 (2.55; 0.7–6.39)	7 (5.19; 2.11–10.39)	0.238
Interaction with the local veterinarian			
Yes	98 (62.42; 54.35–70.01)	91 (67.41; 58.81–75.22)	0.374
No	23 (14.65; 9.52–21.17)	19 (14.07; 8.69–21.1)	0.889
Sometimes	9 (5.73; 2.65–10.6)	7 (5.19; 2.11–10.39)	0.838
None	18 (11.46; 6.94–17.51)	–	<0.001
No answer	9 (5.73; 2.65–10.6)	18 (13.33; 8.1–20.25)	0.025
Reading information on the local notice board			
Yes	99 (63.06; 55–70.61)	73 (54.07; 45.29–62.68)	0.120
No	30 (19.11; 13.28–26.14)	31 (22.96; 16.17–30.98)	0.419
Sometimes	10 (6.37; 3.1–11.4)	7 (5.19; 2.11–10.39)	0.667
None	18 (11.46; 6.94–17.51)	–	0.488
No answer	–	24 (17.78; 11.74–25.29)	0.350
Opinion on the risk of animal-to-human disease transmission			
No risk	2 (1.27; 0.15–4.53)	2 (1.48; 0.18–5.25)	0.879
Acknowledged	76 (48.81; 40.37–56.51)	13 (9.63; 5.23–15.9)	0.000
Avoidable	32 (20.38; 14.38–27.54)	21 (15.56; 9.89–22.79)	0.286
No answer	47 (29.94; 22.9–37.75)	99 (73.33; 65.04–80.57)	<0.001
Availability to personally receive more information about zoonosis from a doctor or veterinarian			
Yes	121 (77.07; 69.7–83.39)	101 (74.81; 66.62–81.89)	0.653
No	18 (11.46; 6.94–17.51)	19 (14.07; 8.69–21.1)	0.504
Does not care	–	1 (0.74; 0.02–4.06)	0.280
No answer	18 (11.46; 6.94–17.51)	14 (10.37; 5.79–16.79)	0.765
Personal history of zoonotic disease (self-reported)			
Yes	7 (4.46; 1.81–8.97)	5 (3.7; 1.21–8.43)	0.746
No	140 (89.17; 83.23–93.56)	126 (93.33; 87.72–96.61)	0.213
Does not know	1 (0.64; 0.02–3.5)	–	0.353
No answer	9 (5.73; 2.65–10.6)	4 (2.96; 0.81–7.41)	0.253
Frequency of hand washing with soap during the day			
2–5 times	64 (40.76; 33–48.88)	41 (30.37; 22.76–38.86)	0.065
6–10 times	27 (17.2; 11.65–24.03)	13 (9.63; 5.23–15.9)	0.061
>10 times	3 (1.91; 0.4–5.48)	14 (10.37; 5.79–16.79)	0.002
As necessary	46 (29.3; 22.32–37.08)	25 (18.52; 12.36–26.11)	0.032
Many times	11 (7.01; 3.55–12.19)	23 (17.04; 11.12–24.46)	0.008
No answer	6 (3.82; 1.42–8.13)	19 (14.07; 8.69–21.1)	0.002
Asking children to wash their hands with soap several times per day			
Yes	127 (80.89; 73.86–86.27)	107 (79.26; 71.44–85.75)	0.727
No	2 (1.27; 0.15–4.53)	–	0.188
No answer	28 (17.83; 12.19–24.73)	28 (20.74; 14.25–28.56)	0.529
Teaching children not to touch dying or dead animals			
Yes	129 (82.17; 75.27–87.81)	105 (77.78; 69.82–84.48)	0.349
No	1 (0.64; 0.02–3.5)	2 (1.48; 0.18–5.25)	0.476
No answer	27 (17.2; 11.65–24.03)	28 (20.74; 14.25–28.56)	0.440

**Table 3:** The distribution of answers to the questions in the second section (10–22).

Item	n (%; 95% CI)		*P*-value (numbers)
	2019	2020	
Breeding of farm animals			
Yes	92 (58.6; 50.47–66.39)	46 (34.07; 26.14–42.72)	<0.001
No	58 (36.94; 29.39–45)	81 (60; 51.22–68.33)	<0.001
No answer	7 (4.46; 1.81–8.97)	8 (5.93; 2.59–11.34)	0.571
Level of sanitary practices in animal husbandry (self-reported) (138 participants)			
None	1 (1.09; 0.03–5.91)	–	0.478
Minimal	4 (4.35; 1.2–10.76)	2 (4.35; 0.53–14.84)	<0.001
Medium	22 (23.91; 15.63–33.94)	32 (69.57; 54.25–82.26)	<0.001
High	14 (15.22; 8.58–24.21)	2 (4.35; 0.53–14.84)	0.060
No answer	51 (55.43; 44.7–65.81)	10 (21.74; 10.95–36.36)	<0.001
Disinfection of animal shelters *			
Yes	136 (86.62; 80.28–91.53)	104 (77.04; 69.02–83.83)	0.033
No	7 (4.46; 1.81–8.97)	3 (2.22; 0.46–6.36)	0.295
Sometimes	–	1 (0.74; 0.02–4.06)	0.280
No answer	14 (8.92; 4.96–14.51)	27 (20; 13.61–27.75)	0.007
Dogs or cats with indoor access			
Yes	36 (22.93; 16.61–30.3)	39 (28.89; 21.42–37.31)	0.245
No	111 (70.7; 62.92–76.68)	84 (62.22; 53.48–70.42)	0.125
No answer	10 (6.37; 3.1–11.4)	12 (8.89; 4.68–15.01)	0.416
Rearing animals in the yard or releasing them outside the household			
Yard	106 (67.52; 59.59–74.76)	72 (53.33; 44.56–61.96)	0.013
Outside	19 (12.1; 7.45–18.25)	4 (2.96; 0.81–7.41)	0.004
Mixed	15 (9.55; 5.45–15.27)	33 (24.44; 17.46–32.58)	0.001
No answer	17 (10.83; 6.44–16.77)	26 (19.26; 12.98–26.93)	0.043
Animal vaccination			
Yes	132 (84.04; 77.4–89.42)	113 (83.7; 76.37–89.5)	0.931
No	13 (8.28; 4.48–13.74)	4 (2.96; 0.81–7.41)	0.053
Sometimes	1 (1.09; 0.03–5.91)	–	0.353
No answer	11 (7.01; 3.55–12.19)	18 (13.33; 8.1–20.25)	0.071
Species vaccinated (245 participants, multiple answers given)			
All	8 (2.74; 1.19–5.33)	27 (13.71; 9.23–19.31)	<0.001
Dogs	112 (38.36; 32.75–44.2)	71 (36.04; 29.34–43.17)	0.604
Cats	36 (12.33; 8.79–16.66)	16 (8.12; 4.71–12.85)	0.139
Poultry	81 (27.74; 22.68–33.26)	40 (20.3; 14.92–26.61)	0.062
Cattle	9 (3.08; 1.42–5.77)	9 (4.57; 2.11–8.5)	0.392
Sheep	8 (2.74; 1.19–5.33)	3 (1.52; 0.32–4.39)	0.373
Goats	2 (0.68; 0.08–2.45)	1 (0.51; 0.01–2.8)	0.805
Horses	5 (1.71; 0.56–3.95)	4 (2.03; 0.56–5.12)	0.797
Pigs	6 (2.05; 0.76–4.42)	3 (1.52; 0.32–4.39)	0.688
Rabbits	4 (1.37; 0.37–3.47)	–	0.099
Bees	–	1 (0.51; 0.01–2.8)	0.223
No answer	21 (7.19; 4.51–10.78)	22 (11.17; 7.13–16.42)	0.128
Treating sick animals by oneself			
Yes	50 (31.85; 24.65–39.75)	22 (16.3; 10.5–23.63)	0.002
No	92 (58.6; 50.47–66.39)	80 (59.26; 50.47–67.63)	0.909
Sometimes	7 (4.46; 1.81–8.97)	16 (11.85; 6.93–18.53)	0.019
No answer	8 (5.1; 2.23–9.79)	17 (12.59; 7.51–19.39)	0.022
Using gloves while treating sick animals			
Yes	55 (35.03; 27.6–43.4)	44 (32.59; 24.78–41.19)	0.661
No	28 (17.83; 12.19–24.73)	19 (14.07; 8.69–21.1)	0.383
Sometimes	1 (1.09; 0.03–5.91)	6 (4.44; 1.65–9.42)	0.034
No answer	73 (46.5; 38.51–54.62)	66 (48.89; 40.19–57.63)	0.683
Measures taken for protecting poultry against avian influenza			
Yes	84 (53.5; 45.38–61.49)	78 (57.78; 48.98–66.22)	0.464
No	45 (28.66; 21.74–36.41)	11 (8.15; 4.14–14.11)	<0.001
No answer	28 (17.83; 12.19–24.73)	46 (34.07; 26.14–42.72)	0.001
Measures taken (162 participants)			
Vaccination	44 (52.38; 41.19–63.4)	24 (30.77; 20.81–42.24)	0.005
Locking in pens	26 (30.95; 21.31–41.98)	23 (29.49; 19.7–40.89)	0.839
Hygiene	13 (15.48; 8.51–25.01)	3 (3.85; 0.8–10.83)	0.013
Other	1 (1.19; 0.03–6.46)	28 (35.9; 25.34–47.56)	<0.001
Undertaken action if animal(s) show(s) signs of disease			
Call to the veterinarian	111 (70.7; 62.92–77.68)	93 (68.89; 60.36–76.57)	0.737
Sacrifice and disposal	16 (10.19; 5.94–16.02)	18 (13.33; 8.1–20.25)	0.404
Sacrifice and consumption	1 (1.09; 0.03–5.91)	1 (0.51; 0.01–2.8)	0.915
Depends on the animal(s)	1 (1.09; 0.03–5.91)	1 (0.51; 0.01–2.8)	0.915
Nothing	2 (1.27; 0.15–4.53)	1 (0.51; 0.01–2.8)	0.652
Other	–	3 (2.22; 0.46–6.36)	0.060
No answer	26 (16.56; 11.11–23.32)	18 (13.33; 8.1–20.25)	0.442
Undertaken action if animal(s) die			
Burial	58 (36.94; 29.39–45)	46 (34.07; 26.14–42.72)	0.610
Incineration	29 (18.47; 12.73–25.44)	16 (11.85; 6.93–18.53)	0.118
Thrown into the trash	6 (3.82; 1.42–8.13)	1 (0.51; 0.01–2.8)	0.086
Call to the veterinarian	37 (23.57; 17.17–30.99)	49 (36.3; 28.2–45.01)	0.017
No answer	27 (17.2; 11.65–24.03)	23 (17.04; 11.12–24.46)	0.971

**Table 4:** The distribution of answers to the questions in the third section (23–29).

Item	n (%; 95% CI)		*P-*value (numbers)
	2019	2020	
Rodents (mice, rats) in the establishment			
Yes	75 (47.77; 39.75–55.88)	63 (46.67; 38.04–55.44)	0.851
No	78 (49.68; 41.61–57.76)	64 (47.41; 38.75–56.18)	0.698
No answer	4 (2.55; 0.7–6.39)	8 (5.93; 2.59–11.34)	0.147
Rodents (mice, rats) inside the house			
Yes	6 (3.82; 1.42–8.13)	5 (3.7; 1.21–8.43)	0.958
Accidentally	1 (0.64; 0.02–3.5)	–	0.353
No	145 (92.36; 87.03–95.09)	119 (88.15; 81.47–93.07)	0.233
No answer	5 (3.18; 1.04–7.28)	11 (8.15; 4.14–14.11)	0,063
Measures against rodents			
Yes	114 (72.61; 64.93–79.42)	92 (68.15; 59.58–75.9)	0.404
No	10 (6.37; 3.1–11.4)	18 (13.33; 8.1–20.25)	0.044
No answer	33 (21.02; 14.93–28.93)	25 (18.52; 12.36–26.11)	0.593
Type of measures against rodents (206 participants, multiple answers given)			
Rodenticides	85 (63.43; 54.68–71.58)	68 (76.4; 66.22–84.76)	0.041
Traps	28 (17.83; 12.19–24.73)	13 (9.63; 5.23–15.9)	0.235
Cats/dogs	18 (11.46; 6.94–17.51)	4 (2.96; 0.81–7.41)	0.028
Glue	1 (1.09; 0.03–5.91)	–	0.414
Ultrasounds	1 (1.09; 0.03–5.91)	2 (4.35; 0.53–14.84)	0.341
No action taken	1 (1.09; 0.03–5.91)	2 (4.35; 0.53–14.84)	0.341
Undertaken action if dying wild animal found			
Call to the veterinarian	44 (28.03; 21.16–35.74)	59 (43.7; 35.19–52.5)	0.005
Notified the authorities	17 (10.83; 6.44–16.77)	19 (14.07; 8.69–21.1)	0.400
Euthanasia	4 (2.55; 0.7–6.39)	1 (0.51; 0.01–2.8)	0.235
Euthanasia + burial	2 (1.27; 0.15–4.53)	–	0.188
Euthanasia + incineration	4 (2.55; 0.7–6.39)	–	0.062
Attempt made to save it	2 (1.27; 0.15–4.53)	4 (2.96; 0.81–7.41)	0.310
Nothing	40 (25.48; 18.87–33.04)	19 (14.07; 8.69–21.1)	0.016
Depends on the animal	1 (1.09; 0.03–5.91)	–	0.353
No answer	43 (27.39; 20.58–35.07)	33 (24.44; 17.46–32.58)	0.568
Undertaken action if dead wild animal found			
Call to the veterinarian	37 (23.57; 17.17–30.99)	42 (31.11; 23.43–39.64)	0.145
Notified the authorities	8 (5.1; 2.23–9.79)	17 (12.59; 7.51–19.39)	0.022
Burial	28 (17.83; 12.19–24.73)	9 (6.67; 3.09–12.28)	0.004
Incineration	6 (3.82; 1.42–8.13)	3 (2.22; 0.46–6.36)	0.430
Thrown into the trash	2 (1.27; 0.15–4.53)	–	0.188
Nothing	34 (21.66; 15.49–28.93)	31 (22.96; 16.17–30.98)	0.789
Depends on the animal	3 (1.91; 0.4–5.48)	–	0.106
No answer	39 (24.84; 18.3–32.36)	33 (24.44; 17.46–32.58)	0.938
Hunter			
Yes	7 (4.46; 1.81–8.97)	6 (4.44; 1.65–9.42)	0.995
No	150 (95.54; 91.03–98.19)	127 (94.07; 88.66–97.41)	0.571
No answer	–	2 (4.35; 0.53–14.84)	0.126

**Table 5:** The distribution of answers to the questions in the fourth section (32–38).

Item	n (%; 95% CI)		P value(Numbers)
	2019	2020	
Trichinoscopy performed on sacrificed pigs’ meat			
Yes	68 (43.31; 35.44–51.45)	54 (40; 31.67–48.78)	0.567
No	54 (34.39; 27.01–42.39)	28 (20.74; 14.25–28.56)	0.010
Sometimes	2 (1.27; 0.15–4.53)	2 (4.35; 0.53–14.84)	0.879
No answer	33 (21.02; 14.93–28.93)	51 (37.78; 29.58–46.52)	0.002
Fish consumption (times/week)			
Rarely	6 (3.82; 1.42–8.13)	7 (5.19; 2.11–10.39)	0.573
<1 time	11 (7.01; 3.55–12.19)	7 (5.19; 2.11–10.39)	0.519
1 time	41 (26.11; 19.44–33.72)	42 (31.11; 23.43–39.64)	0.345
2 times	34 (21.66; 15.49–28.93)	21 (15.56; 9.89–22.79)	0.184
3 times	16 (10.19; 5.94–16.02)	5 (3.7; 1.21–8.43)	0.032
4 times	8 (5.1; 2.23–9.79)	2 (4.35; 0.53–14.84)	0.090
5 times	–	2 (4.35; 0.53–14.84)	0.126
Very often	3 (1.91; 0.4–5.48)	2 (4.35; 0.53–14.84)	0.778
Depends	25 (15.92; 10.58–22.6)	–	<0.001
No answer	13 (8.28; 4.48–13.74)	47 (34.81; 26.83–43.49)	<0.001
Consumption of raw fish or fish parts from the DDBR			
Yes	8 (5.1; 2.23–9.79)	11 (8.15; 4.14–14.11)	0.292
No	140 (89.17; 83.23–93.56)	116 (85.93; 78.9–91.31)	0.400
No answer	9 (5.73; 2.65–10.6)	8 (5.93; 2.59–11.34)	0.944
Consumption of undercooked fish from the DDBR			
Yes	17 (10.83; 6.44–16.77)	20 (14.81; 9.29–21.95)	0.307
No	128 (81.53; 74.56–87.27)	101 (74.81; 66.62–81.89)	0.164
No answer	12 (7.64; 4.01–12.97)	14 (10.37; 5.79–16.79)	0.415
Seen fish (perch, zander) with wireworms (*Eustrongylides* sp.)			
Yes	94 (59.87; 51.76–67.6)	63 (46.67; 38.04–55.44)	0.024
No	58 (36.94; 29.39–45)	68 (50.37; 41.64–59.08)	0.021
Does not know	1 (0.64; 0.02–3.5)	–	0.353
No answer	4 (2.55; 0.7–6.39)	4 (2.96; 0.81–7.41)	0.828
Consumption of fish with wireworms (*Eustrongylides* sp.)			
Yes	46 (29.3; 22.32–37.08)	25 (18.52; 12.36–26.11)	0.032
No	96 (61.15; 53.05–68.81)	73 (54.07; 45.29–62.68)	0.222
No answer	15 (9.55; 5.45–15.27)	37 (27.41; 20.09–35.75)	<0.001
Details…(71 participants, multiple answers given)			
Boiled	19 (50; 33.38–66.62)	14 (36.84; 21.81–54.01)	0.247
Fried	10 (26.32; 13.4–43.1)	12 (31.58; 17.5–48.65)	0.613
Worms removed	4 (10.53; 2.94–24.8)	6 (15.79; 6.02–31.25)	0.497
Thermal processing	5 (13.16; 4.41–28.09)	6 (15.79; 6.02–31.25)	0.744

**Table 6:** The distribution of answers to the questions in the fifth section (39–45).

Item	n (%; 95% CI)		*P-*value (numbers)
	2019	2020	
Fear of ticks			
Yes	70 (44.59; 36.66–52.72)	65 (48.15; 39.47–56.91)	0.543
No	85 (63.43; 54.68–71.58)	68 (50.37; 41.64–59.08)	0.520
No answer	2 (1.27; 0.15–4.53)	2 (4.35; 0.53–14.84)	0.879
Walking in areas with risk of tick bites			
Yes	102 (64.97; 56.96–72.4)	80 (59.26; 50.47–67.63)	0.315
No	50 (31.85; 24.65–39.75)	45 (33.33; 25.46–41.96)	0.787
Sometimes	1 (0.64; 0.02–3.5)	3 (2.22; 0.46–6.36)	0.245
Rarely	1 (0.64; 0.02–3.5)	4 (2.96; 0.81–7.41)	0.127
No answer	3 (1.91; 0.4–5.48)	3 (2.22; 0.46–6.36)	0.852
Ticks found on animals			
Yes	112 (71.34; 63.59–78.26)	106 (78.52; 70.63–85.12)	0.160
No	40 (25.48; 18.87–33.04)	17 (12.59; 7.51–19.39)	0.006
No answer	5 (3.18; 1.04–7.28)	12 (8.89; 4.68–15.01)	0.036
Animal species (218 participants, multiple answers given)			
Dogs	96 (59.63; 51.62–67.28)	70 (48.95; 40.51–57.44)	0.062
Cats	9 (5.59; 2.59–10.35)	21 (14.69; 9.33–21.57)	0.008
Poultry	1 (0.62; 0.02–3.41)	1 (0.7; 0.02–3.83)	0.933
Cattle	19 (11.8; 7.26–17.81)	17 (11.89; 7.08–18.35)	0.981
Sheep	11 (6.83; 3.46–11.9)	23 (16.08; 10.48–23.15)	0.011
Goats	4 (2.48; 0.68–6.24)	7 (4.9; 1.99–9.83)	0.261
Horses	8 (4.97; 2.17–9.56)	3 (2.1; 0.43–6.01)	0.181
Pigs	10 (6.21; 3.01–11.13)	–	0.002
Rabbits	1 (0.62; 0.02–3.41)	–	0.345
Boars	1 (0.62; 0.02–3.41)	–	0.345
Tortoises	1 (0.62; 0.02–3.41)	1 (0.7; 0.02–3.83)	0.933
Tick protection/removal actions for animals			
Yes	119 (75.8; 68.33–82.27)	109 (80.74; 73.07–87.02)	0.309
No	18 (11.46; 6.94–17.51)	8 (5.93; 2.59–11.34)	0.098
No answer	20 (12.74; 7.96–18.99)	18 (13.33; 8.1–20.25)	0.880
Details…(219 participants)			
Mechanical removal	18 (19.78; 12.16–29.45)	24 (18.75; 12.4–26.6)	0.849
Chemicals	67 (73.63; 63.35–82.31)	83 (64.84; 55.91–73.07)	0.168
Other	6 (6.59; 2.46–13.8)	21 (16.41; 10.45–23.98)	0.029
Removing ticks by hand without wearing gloves			
Yes	33 (21.02; 14.93–28.93)	16 (11.85; 6.93–18.53)	0.037
No	80 (50.96; 42.86–59.01)	92 (68.15; 59.58–75.9)	0.003
No answer	44 (28.03; 21.16–35.74)	27 (20; 13.61–27.75)	0.111

We chose to address the investigations regarding the percentages obtained in this study, even though the analysis for statistical significance (*p*-value) was performed using numbers to make it simpler to understand the significance of the results obtained.

## Results

**Figure 1:**
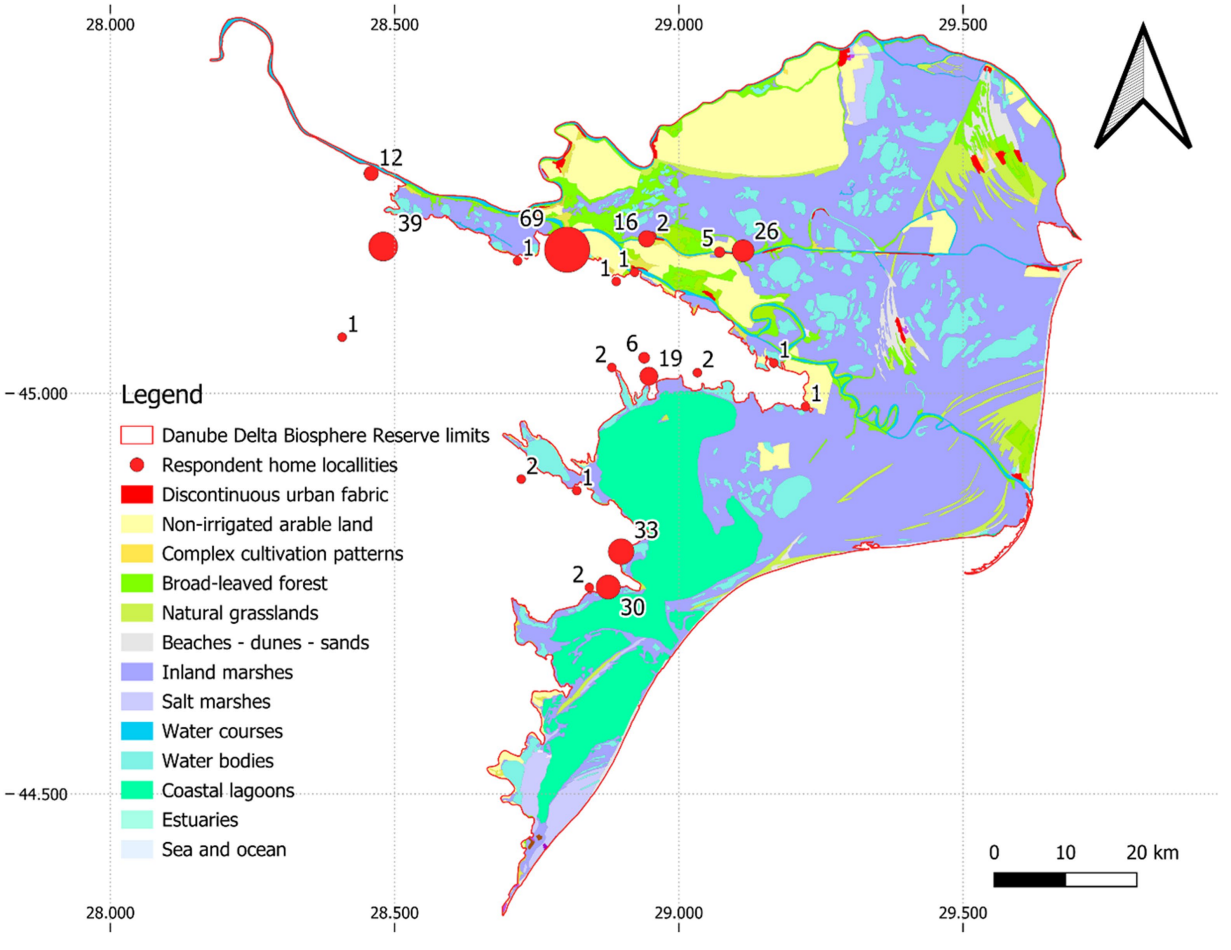
Distribution on the map of the number of respondents interviewed/locality.

A total of 292 adults participated in our questionnaires during both study years, of which 6 respondents came from extra-county areas, but were present in the target locations at the time of the 2020 survey (Table 1; Figure 1).

A total of 157 respondents were interviewed in 2019, and 135 in 2020; the lower sample size in 2020 likely reflects movement restrictions related to COVID-19. The average age of the participants was 47.2 ± 14.8 years in 2019 and 42.3 ± 18.2 years in 2020, with medians of 49 years in 2019 and 48 years in 2020, respectively.

### Background information

#### Employment status and relationship with veterinarians

In 2019, 17.83% of respondents reported being unemployed; this figure decreased to 7.41% in 2020. The proportion of non-respondents in this category rose from 2.55 to 5.19% in 2020. Similarly, the percentage of identified retirees increased from 7.64 to 11.85% in 2020. The majority of the remaining respondents fell into broader categories related to agriculture and animal husbandry, comprising 23.57% in 2019 and 30.37% in 2020.

**Figure 2:**
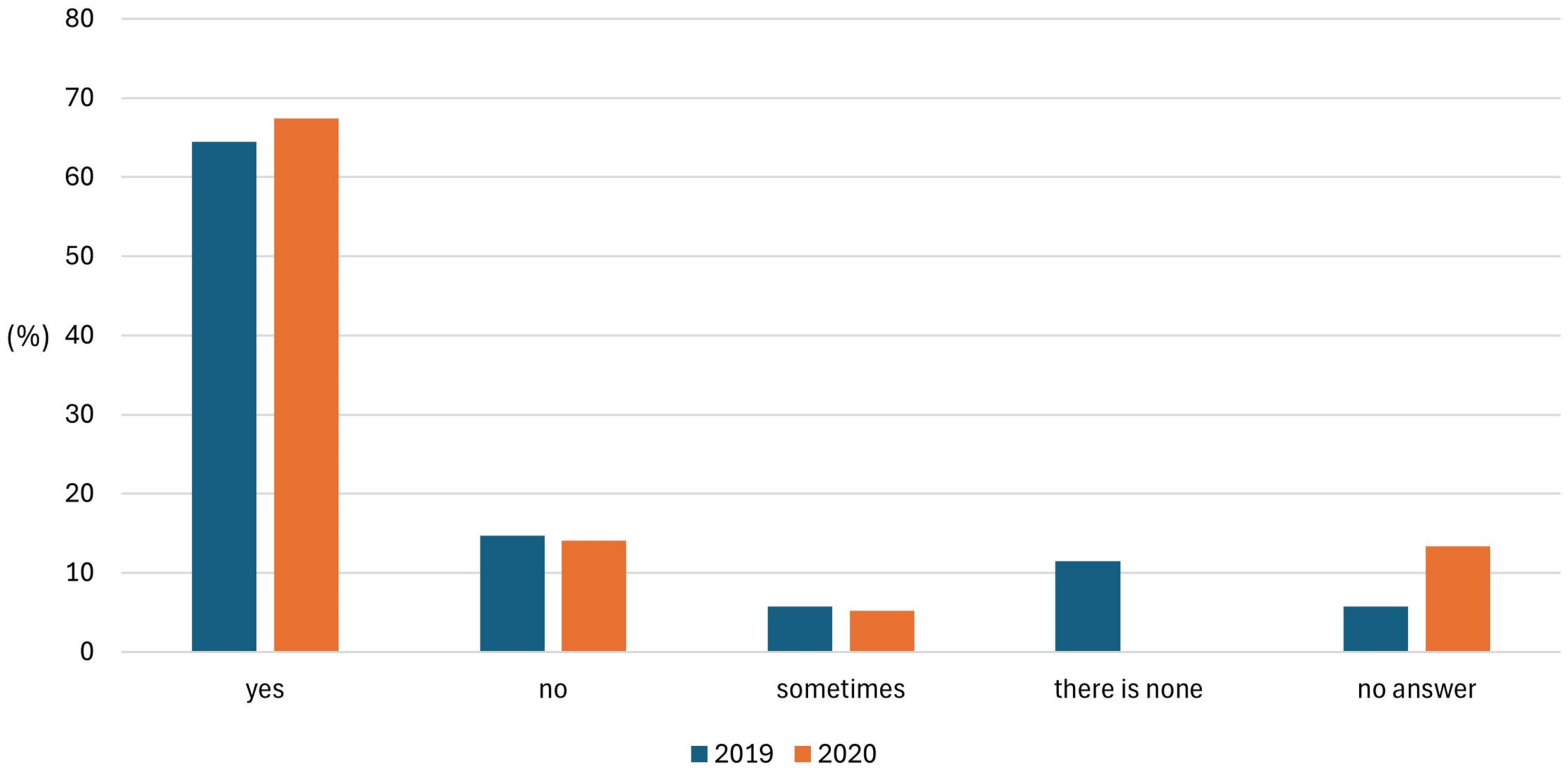
Interaction with the local veterinarian.

Regarding professional interactions, 62.42% of participants reported frequent collaboration with local veterinarians in 2019, with a slight increase to 67.41% in 2020, as presented in Figure 2 (*p* ≈ 0.561) (Table 2).

**Figure 3:**
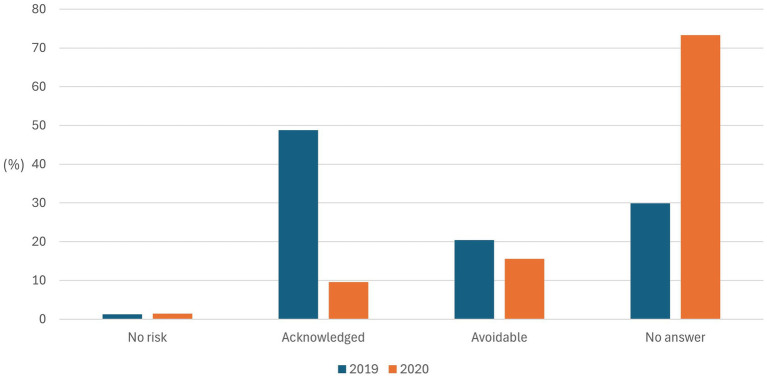
Opinion on the risk of animal-to-human disease transmission.

Regarding reading information on local boards, a decline was observed from 63.06% in 2019 to 54.07% in 2020 (*p* = approx. 0.32658), a trend likely influenced by movement restrictions because of COVID-19. Figure 3 shows that 48.81% of respondents initially acknowledged the risk of disease transmission from animals to humans, which decreased to 9.63% the following year (*p* ≈ 0.01). Although unexpected, this decline may indicate potential public rejection of measures related to the evolution of the COVID-19 pandemic.

#### Personal hygiene

**Figure 4:**
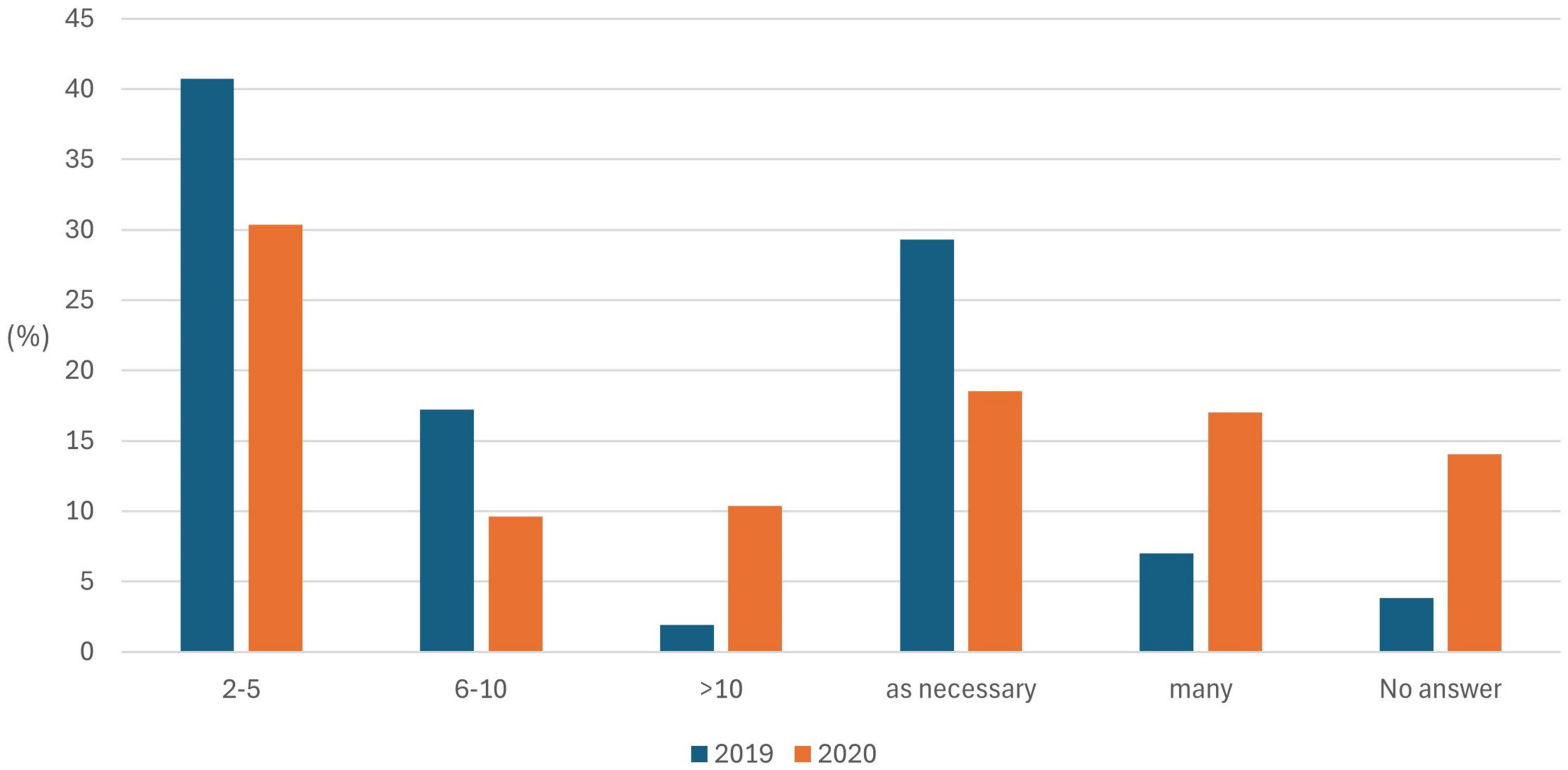
Frequency of hand washing with soap during the day.

For individuals who reported washing their hands more than 10 times per day (Figure 4), the percentage increased from 1.91 to 10.37% in 2020, with a statistically significant *p*-value of approximately 0.0035. Although the frequency of handwashing with soap 2–5 times/day showed some decline, from 40.76 to 30.37% in 2020, the percentage of individuals who viewed this practice as important remained significant.

Additionally, among those who reported washing their hands multiple times daily, the difference between the 2 years was statistically significant, rising from 7.01 to 17.04% in 2020, with a *p*-value of approximately 0.0163. However, the percentage of respondents who washed their hands frequently remained quite low, even when including those who answered “whenever necessary” or “often.”

A substantial majority of respondents (80.89% in 2020, up from 79.26% in 2019) reported encouraging their children to wash their hands multiple times a day. Similarly, 82.17% in 2019 and 77.78% in 2020 advised children to avoid contact with dead or dying animals.

#### Domestic animals

Table 3 summarizes responses regarding animal husbandry, highlighting marked differences over the study period.

The proportion of participants identifying as livestock breeders dropped significantly from approximately 58.6% in 2019 to 34% in 2020. However, among these farmers, the perception of hygiene measures as having medium to high effectiveness rose significantly from 39.13% in 2019 to 73.92% in 2020 (*p* ≈ 0.0016). Of note, it is concerning that a large number of respondents did not answer this question.

**Figure 5:**
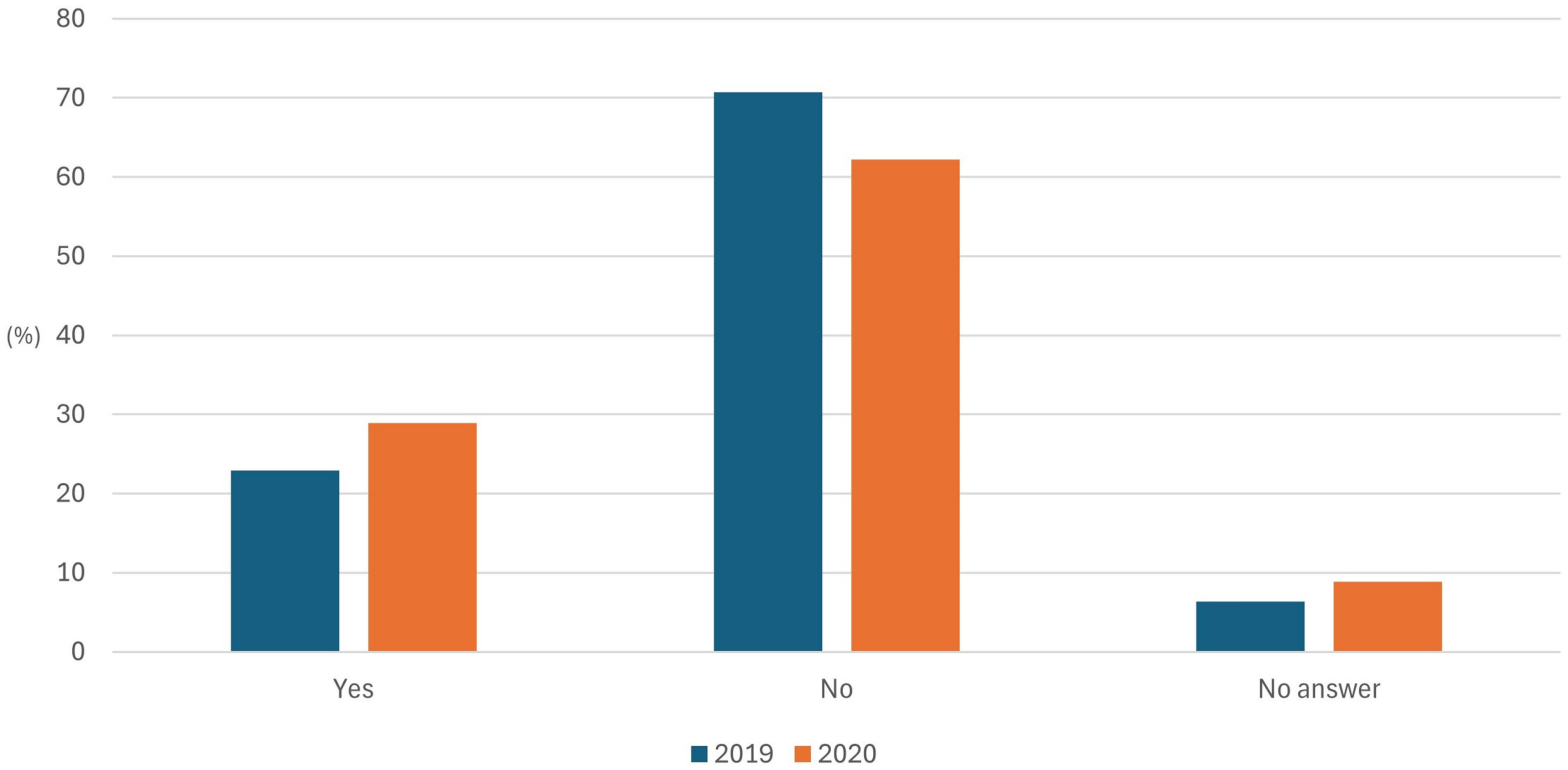
Dogs or cats with indoor access.

A high percentage of respondents confirmed the regular disinfection of animal shelters (86.62% in 2019 and 77.04% in 2020). Meanwhile, the percentage of domestic carnivores who were allowed into the home increased significantly from 22.93% in 2019 to 28.89% in 2020 (Figure 5) (*p* ≈ 0.011).

The majority of the participants kept animals in their backyards (67.52% in 2019), a practice that decreased to 53.32% in 2020. Although this difference was not statistically significant (*p* ≈ 0.132), the downward trend may be attributed to pandemic-related movement restrictions. While the reported use of free-range or mixed systems increased from 21.65% in 2019 to 27.40% in 2020, the percentage of non-responses also rose from 10.83 to 19.26% in 2020. This likely indicates a reporting bias, with participants likely to have been reluctant to disclose non-compliant free-range practices. Therefore, it is estimated that the overall percentage of those using mixed or free-range systems, plus all those who did not want to answer, increased from 32.48 to 46.67% in 2020 (*p* ≈ 0.046). This shift may be explained by mobility restrictions due to the COVID-19 pandemic and local conditions that necessitated semi-free breeding methods.

Vaccination rates remained stable, with 84.04 and 83.7% of participants reporting animal vaccinations in 2019 and 2020, respectively. However, veterinary consultations for sick animals saw a slight decline from 70.7 to 68.89% in 2020, likely due to circulation restrictions. The percentage of participants who applied treatments to animals themselves decreased from 31.85 to 16.3% in 2020. Notably, the use of protective measures (such as gloves) when treating sick animals decreased from 35.03 to 32.59% in 2020, while the percentage of those employing no protective measures decreased from 17.83 to 14.07% in 2020.

Awareness of avian influenza protection increased, with 53.5% of participants taking measures in 2019 compared to 57.78% in 2020.

Regarding dead animals, preferences for burial or cremation decreased from 55.4 to 45.92% in 2020. Disposing of them in the trash decreased from 3.82 to 0.51% in 2020, while contacting a veterinarian increased from 23.57 to 36.3% (*p* ≈ 0.050).

### Risks associated with wildlife

Table 4 summarizes responses regarding risks associated with wildlife.

Rodent presence was reported in 46.67% of households in 2020, consistent with the previously reported 47.77%. The percentage of respondents who stated that rodents did not enter their homes remained high (92.36% in 2019; 88.15% in 2020). Approximately 72.61 and 68.15% of participants, respectively, implemented anti-rodent measures. The use of rodenticides increased from 63.43% in 2019 to 76.4% in 2020, while reliance on biological controls (cats and dogs) decreased from 11.46 to 2.96% in 2020.

**Figure 6:**
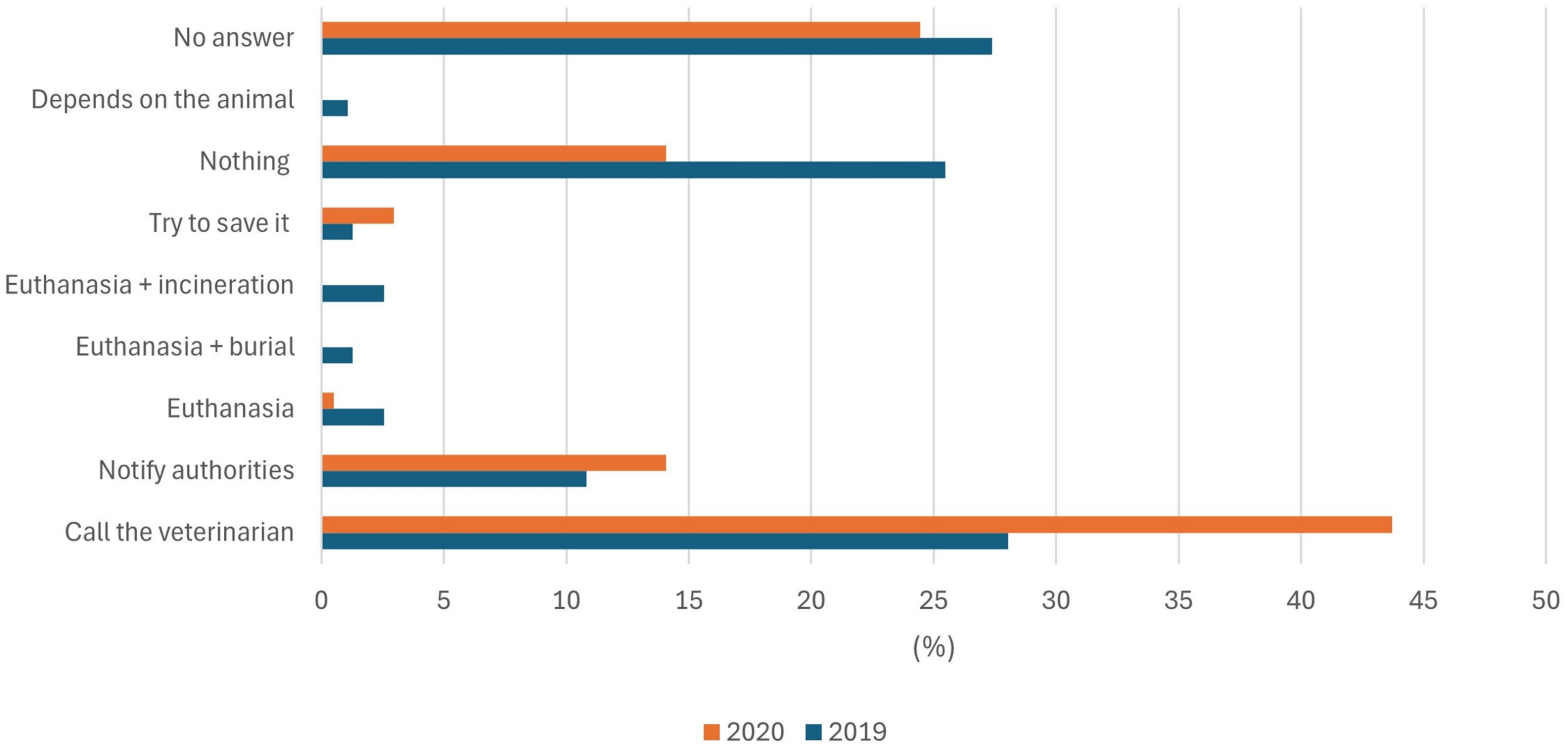
Undertaken action if a dying wild animal is found.

**Figure 7:**
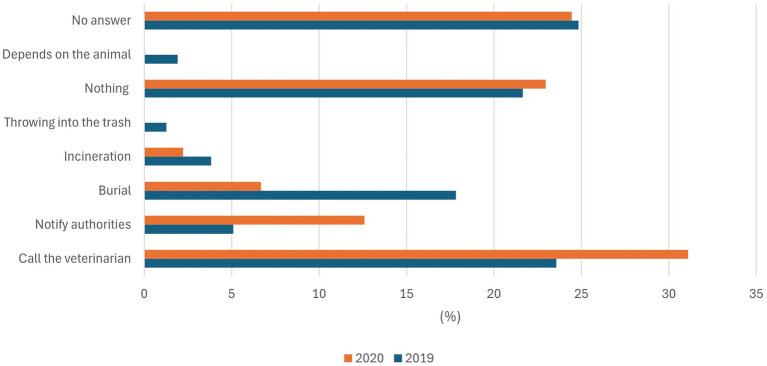
Undertaken action if a dead wild animal is found.

In 2020, a significantly higher percentage of respondents said they would notify a veterinarian upon encountering a dying wild animal (43.7% vs. 28.03% in 2019; *p* ≈ 0.038) (Figure 6), while a lower percentage said they would ignore it (14.07% vs. 25.48%). The percent of respondents who would report dead wild animals to authorities or veterinarians also increased (43.7% vs. 28.67%), with a slight rise in those who would ignore carcasses (22.96% vs. 21.66%) (Figure 7).

### Foodborne pathogens

Table 5 records the changes in foodborne pathogen practices between 2019 and 2020.

Sample collection for *Trichinella* spp. testing decreased from 43.31 to 40.31%.

Consumption of fish (more than once weekly) ~ dropped from 63.06% in 2019 to 51.72% in 2020, while consumption of raw fish remained low (5.1 and 8.15%, respectively).

Observations of *Eustrongylides* spp. in fish decreased from 59.87 to 46.67%. The percentage of respondents consuming infested fish without concern decreased from 29.3 to 18.52% in 2020. The majority of consumers who found parasites either removed them or cooked the fish.

### Zoonoses and ticks

In Table 6, some observations were recorded regarding tick-borne diseases, which showed a slight upward trend, with fear of ticks increasing from 44.59 to 48.15% in 2020. The frequency of visiting areas with dense vegetation decreased slightly from 64.97 to 59.26% in 2020. However, reports of ticks on animals increased (71.34% in 2019 vs. 78.52% in 2020), with dogs, sheep, and cattle being the most affected species.

Proactive tick management increased, with 80.74% of participants taking measures in 2020 compared to 75.8% the previous year. Furthermore, the frequency of unsafe tick removal (without gloves) decreased notably from 21.02% in 2019 to 11.85% in 2020.

## Discussion

Across waves, self-reported hygiene practices and preventive actions were common, but not everyone did so consistently in all areas of their lives, reflecting patterns in the literature that show high adherence in some contexts while highlighting persistent gaps in risk-related behaviors, particularly among rural and semi-rural populations with varying access to resources and communication channels. This inconsistency undermines efforts to prevent the spread of illnesses and establish consistent healthy behaviors across all populations, which are clear objectives of the relevant authorities.

Personal hygiene, including that of children, was generally not neglected, with participants often taking precautions against potential infections through self-protection and protective measures even for their animals. However, despite majority compliance with sanitary and preventive measures, a significant proportion of respondents showed risk-related behaviors, underscoring the need for targeted interventions.

Given that the 2019 and 2020 samples comprise distinct cohorts, and respondents’ behaviors may have been influenced by exposure to disruptive public information (e.g., restrictions on human and animal movement, trade alterations, and culling due to disease), the observed differences cannot be attributed solely to the evolution of the COVID-19 pandemic. Therefore, we present the results of the questionnaire with cautious interpretations, as a superficial reading could otherwise lead to conclusions that may not exactly correspond to reality. Still, it is reasonable to conclude that all the information about hygiene and the rules about staying at home likely had an impact on individuals’ hygiene habits, potentially changing some broader habits and ways of thinking.

Interpretation of the first-wave responses indicates that participants were, on average, relatively well informed about zoonoses and transmission pathways, showing receptivity to guidance from medical and veterinary authorities. The pronounced reduction in affirmative responses in the following year, along with increased non-responses to questions on perceived zoonotic risks, suggests a decline in awareness and raises doubts about the efficacy of risk communication strategies during and before the study period.

On the other hand, this could also have been caused by the methodological limitations of the questionnaire and a different respondent base in the following year. However, we can conclude that the results of the first year did not accurately reflect the situation, and more detailed studies, with a more in-depth, specific sociological model, need to be organized and applied to reduce biases.

The percentage of respondents who did not answer the question about using veterinary services increased substantially in 2020, and the evolution of the COVID-19 pandemic was not the only cause. In this situation, it can be assumed that this reflects respondents’ reluctance to disclose non-use of veterinary services; however, since this is not certain, no definitive statements can be made. In addition, when talking about veterinarians, in the future a distinction will have to be made between state and private veterinary services, especially in the context of the evolution of numerous outbreaks of emerging diseases in domestic animals, which have required the culling of a large number of animals, which has also greatly affected the material condition of their owners and may have negatively influenced public perception of official veterinary services.

Four key points emerge regarding domestic animal health:

A potentially overlooked risk is the practice of allowing animals to roam freely, reported by 24.3% of participants, which heightens pathogen exposure through contact with other domestic or wild animals. For instance, the 2018 African Swine Fever epidemic in Tulcea County was partly linked to wild boars (13). Similarly, free-range ducks and geese in the Danube Delta Biosphere Reserve (DDBR) tested positive for avian influenza without showing symptoms, acting as reservoirs for disease spread (14).

Refusal to vaccinate the animals was acknowledged by 8.28% of the participants in 2019 and 2.96% in 2020. If we add the percentages of non-responses (7.01% in 2019 and 13, 3 % in 2020), which can also be interpreted as refusals (even if partial, and only for certain species of animals), the results are concerning. This is especially true given the mandatory vaccinations in Romania for anthrax in herbivores, rabies in carnivores, and Newcastle disease in poultry (Romanian Government Decision No. 1,156, 2013) (30) and even more so in the Danube Delta region where numerous outbreaks of anthrax, avian influenza, Newcastle disease, and rabies have occurred over time.

While the majority of participants reported consulting veterinarians for sick animals, some respondents self-treated or culled animals, potentially leading to bacterial infections such as Salmonella or *Escherichia coli* through improper handling (15, 16).

The improper disposal of dead animals, such as abandoning carcasses in fields or water bodies for scavengers, persists and poses a risk of disseminating infectious agents in areas with inadequate waste management.

Regarding wildlife contact, the widespread presence of rodents was acknowledged, but not all participants used effective control methods. Reliance on cats and dogs for rodent control can inadvertently spread parasites such as Toxoplasma gondii, which affects humans and animals worldwide (17, 18) and can have severe consequences (28, 33), for example during pregnancy (19). Rodents are involved in the biological cycle of some parasites and can serve as hosts for pathogens, including cestodes, *Trichinella* spp. (20, 27), Hantavirus [not yet in Romania; (21)], and some bacteria, such as *Leptospira* spp. (16).

For encounters with dying or dead wild animals that may carry zoonotic agents, notifying veterinarians or authorities is the recommended approach; however, ignoring such findings, as reported by many participants, poses indirect risks to other species.

Regarding foodborne pathogens, despite legislation requiring *Trichinella* spp. inspections, some individuals are known to bypass veterinary checks, heightening infection risks (22, 31). In the study area, fish consumption-related risks were prominent, with raw or undercooked fish linked to zoonotic agents (23). Awareness of parasites such as *Eustrongylides* spp., which were first studied in the Danube Delta (24), is mixed, as 25% of participants admitted to consuming them, although proper cooking mitigates the risk (25, 26).

Many individuals who took part in the study found ticks on animals and used chemicals to protect them, which is very important because ticks in Romania can carry diseases that can be passed from animals to humans. Researchers such as Borșan, Raileanu, Mărcuțan, Matei, and Kalmar have shown that ticks in Romania can carry numerous diseases. By protecting animals from ticks, we can also help keep humans healthy (29, 34–37).

For some questionnaire items, no statistically significant correlations emerged, although improvements in hygiene adoption were noted. Significant correlations were found for items such as recognizing zoonotic risks, hand-washing frequency, and notifying veterinarians, indicating positive shifts potentially due to warning measures.

Notably, the majority of those interviewed, regardless of their profession, were found to own several or more species of animals, of course with variations in their number. However, factors such as fear of authorities, lack of trust, or misunderstanding of the questionnaire contributed to incomplete responses. This partly explains the increase in agricultural employment (23.57% in 2019 to 30.37% in 2020) alongside a decline in self-identification as animal breeders (58.6–34%), as some owners viewed their subsistence farms as non-professional.

These insights enhance our understanding of prevention strategies, emphasizing the role of trusted local channels in risk communication, consistent with evidence on rural populations.

## Conclusion

This study revealed that residents in and around the Danube Delta Biosphere Reserve (DDBR) possess a baseline knowledge of zoonoses and engage in preventive practices, although persistent gaps in risk perception and hygiene behaviors remain. The COVID-19 pandemic amplified hygiene messaging, but it also exposed challenges in information uptake and trust in authorities, as evidenced by our two-wave survey, which provided snapshots of the situation without establishing causality.

Analysis of response trends across surveys highlighted increases in awareness and precautions in several areas; however, ongoing risky behaviors underscore the need for sustained educational efforts amid evolving threats.

Inconclusive responses suggest that awareness of hygiene and risk reduction has not fully taken root, necessitating multifaceted campaigns that use media and, most importantly, direct communication to address these gaps.

The pandemic’s influence on personal hygiene is clear, but in regions such as the study area, cognitive dissonance and psychological reactance—likely fueled by misinformation and declining trust in institutions—led to contradictory responses, emphasizing the limitations of emergency messaging.

Targeted, culturally appropriate interventions are required to improve preparedness. Multisectoral collaboration among veterinarians, public health professionals, and environmental experts is essential to deliver clear, context-sensitive messages.

Future studies should employ repeated longitudinal or cross-sectional survey designs with consistent sampling to minimize errors and strengthen causal inferences where possible, helping relevant authorities improve their interventions.

## Data Availability

The raw data supporting the conclusions of this article will be made available by the authors, without undue reservation.
